# Supplementary figures and images for: Host kinase regulation of Plasmodium vivax dormant and replicating liver stages
Source: PLoS Negl Trop Dis. 2026 Feb 25;20(2):e0014053. doi: 10.1371/journal.pntd.0014053 (PMC12959719; doi:10.1371/journal.pntd.0014053)

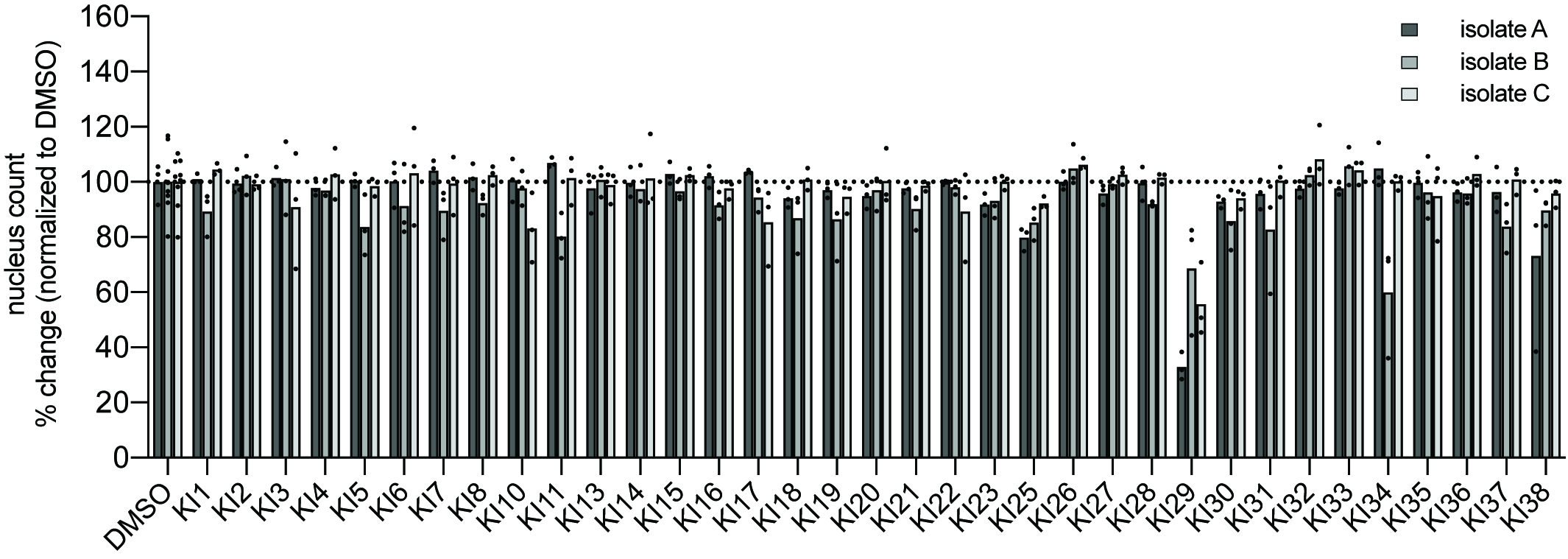

Supplement: S1 Fig — Primary hepatocyte nuclei were quantified in each infected well at the time of parasite quantification, 8 days post-infection, as a measure of kinase inhibitor toxicity. Each dot represents a technical replicate. N = 3–9. Data were normalized to the DMSO control average for each isolate and analyzed by Dunnett multiple comparisons test. (TIF) [file pntd.0014053.s001.tif]

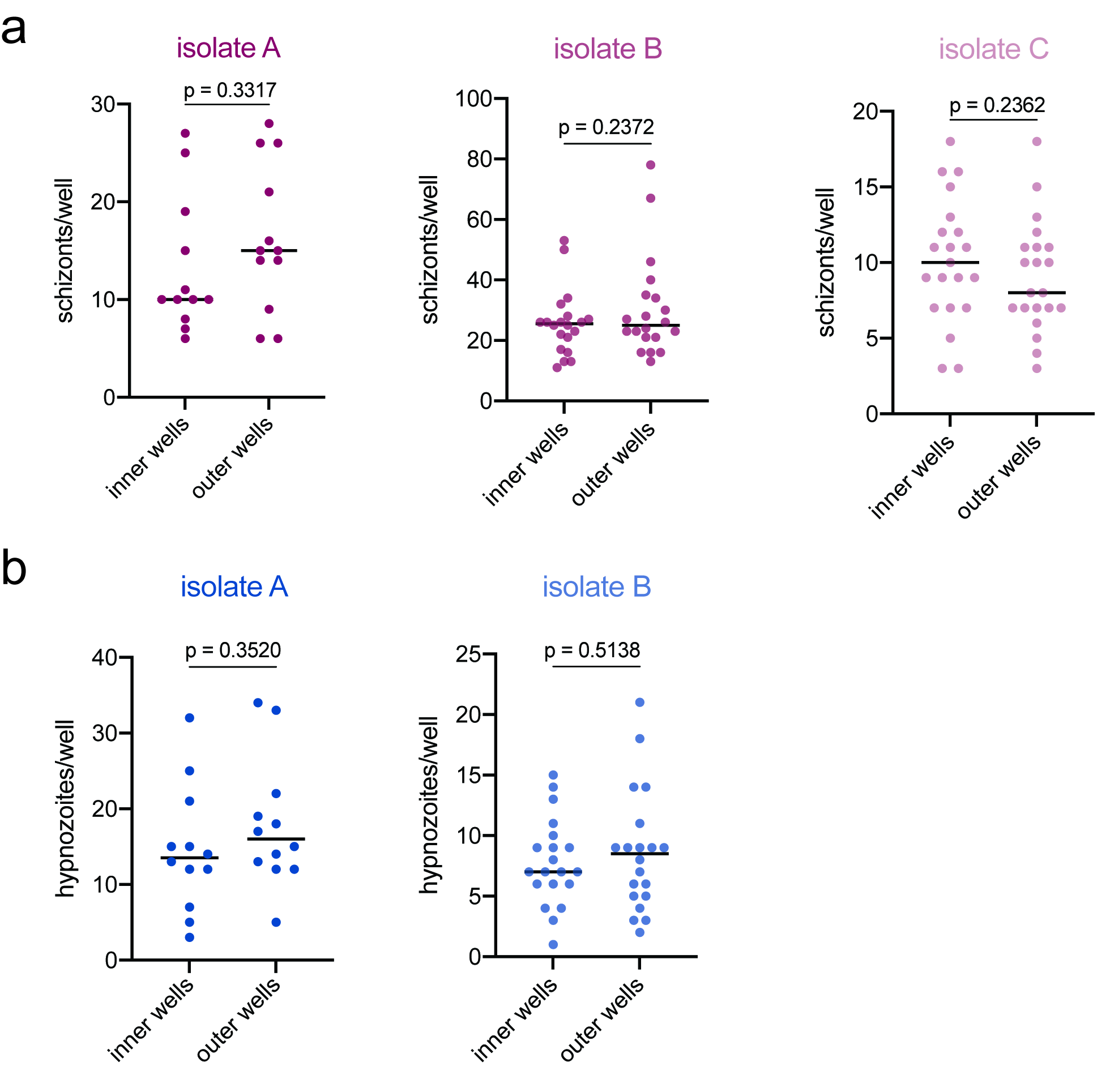

Supplement: S2 Fig — (a) Schizont and (b) hypnozoite numbers were compared between outer edge (D5-15, L5-15) and inner (F5-15, J5-15) wells in each 384-well plate (one plate per isolate) that received the same treatment. Each dot represents counts from a single well. Data were analyzed by paired t-test. (TIF) [file pntd.0014053.s002.tif]

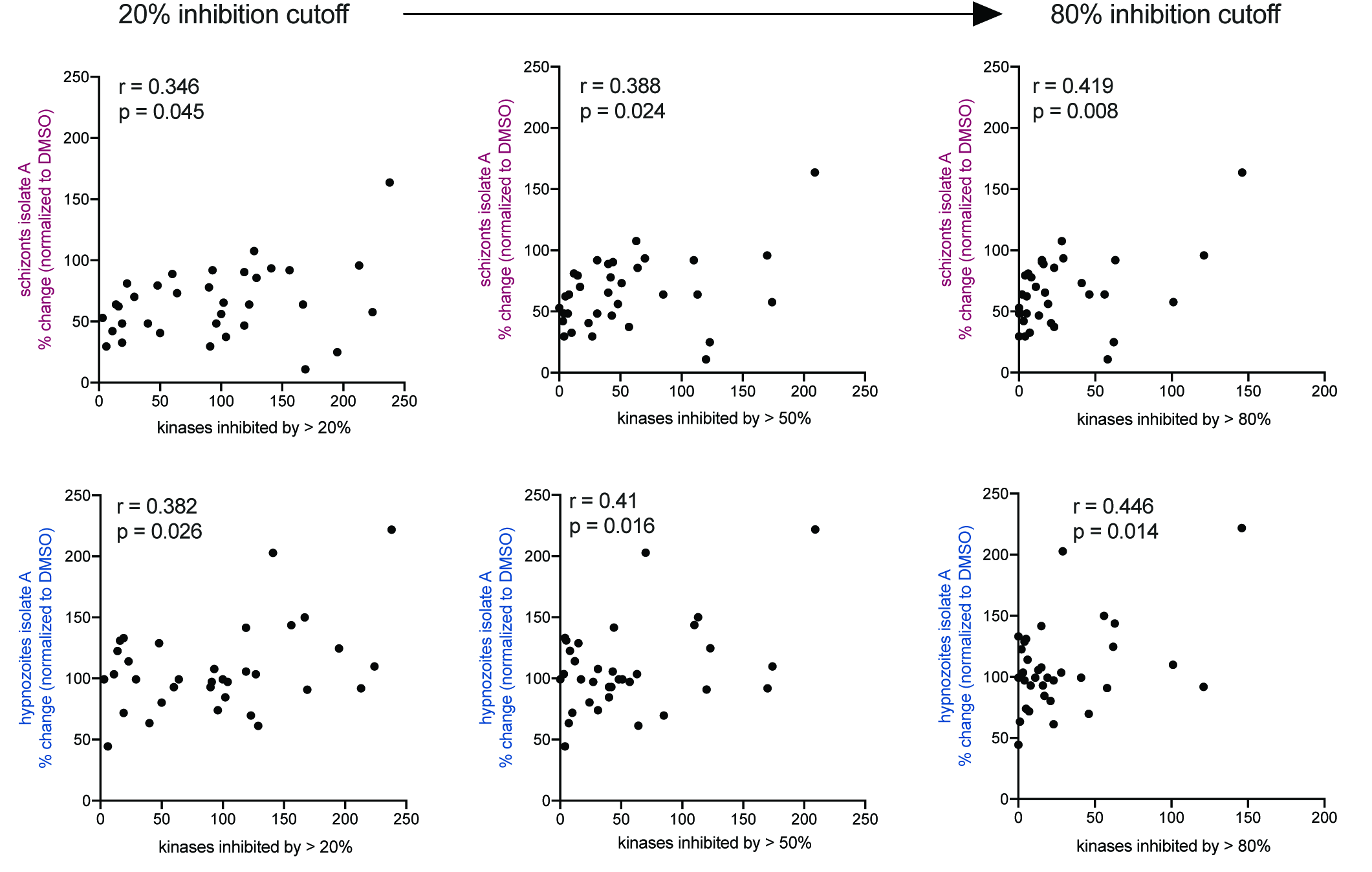

Supplement: S3 Fig — The effect of each inhibitor on schizont or hypnozoite numbers, for isolate A, is plotted against the number of kinases inhibited by each inhibitor using cut-offs of 20%, 50% or 80%. Each dot represents a single inhibitor. Pearson correlation coefficients (r) and p-values (p) were calculated for each plot. (TIF) [file pntd.0014053.s003.tif]

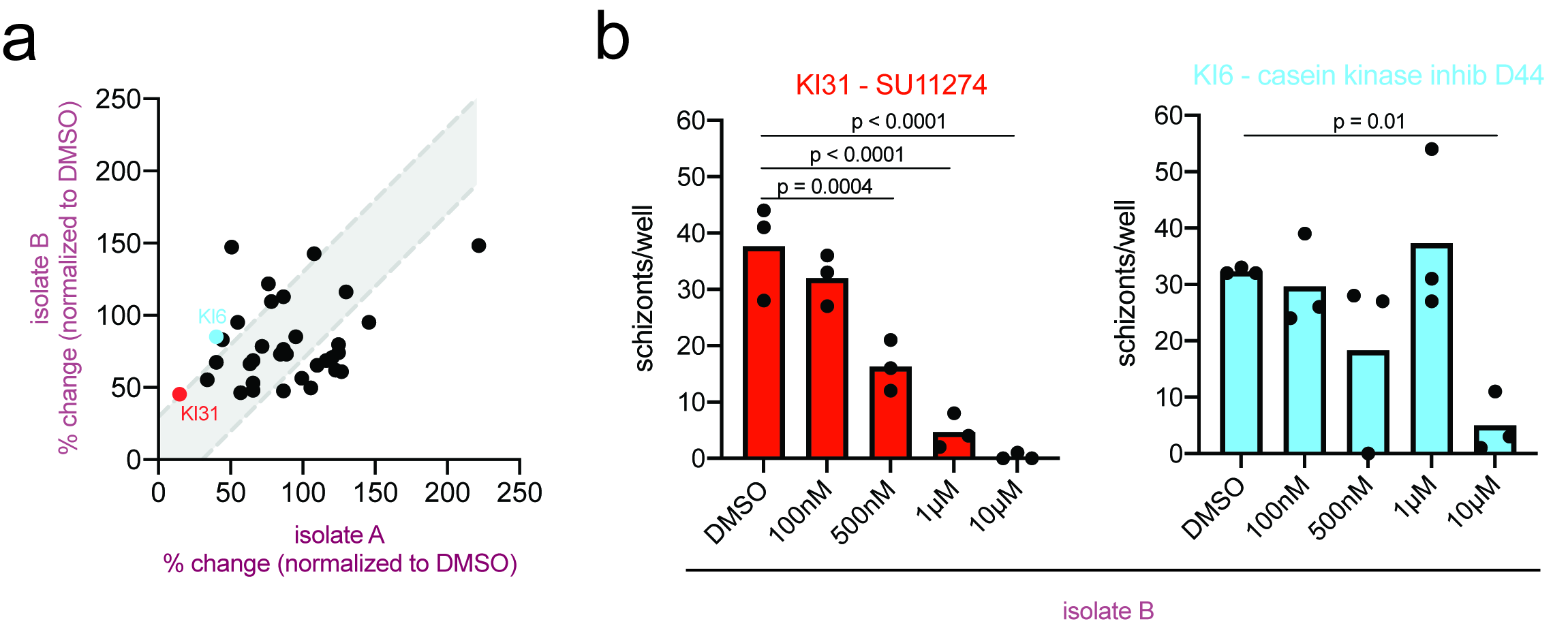

Supplement: S4 Fig — (a) Correlation between effect of kinase inhibitors on the number of schizonts, normalized to DMSO controls, in isolate A vs isolate B. Gray shaded area indicates a 95% confidence interval, based on the variation of control wells, around x = y. Each dot represents an inhibitor. Inhibitors for which dose response curves were done are highlighted. (b) Schizont dose response curves for two kinase inhibitors. Each dot represents a technical replicate. Data were analyzed by Fisher’s LSD test. (TIF) [file pntd.0014053.s004.tif]

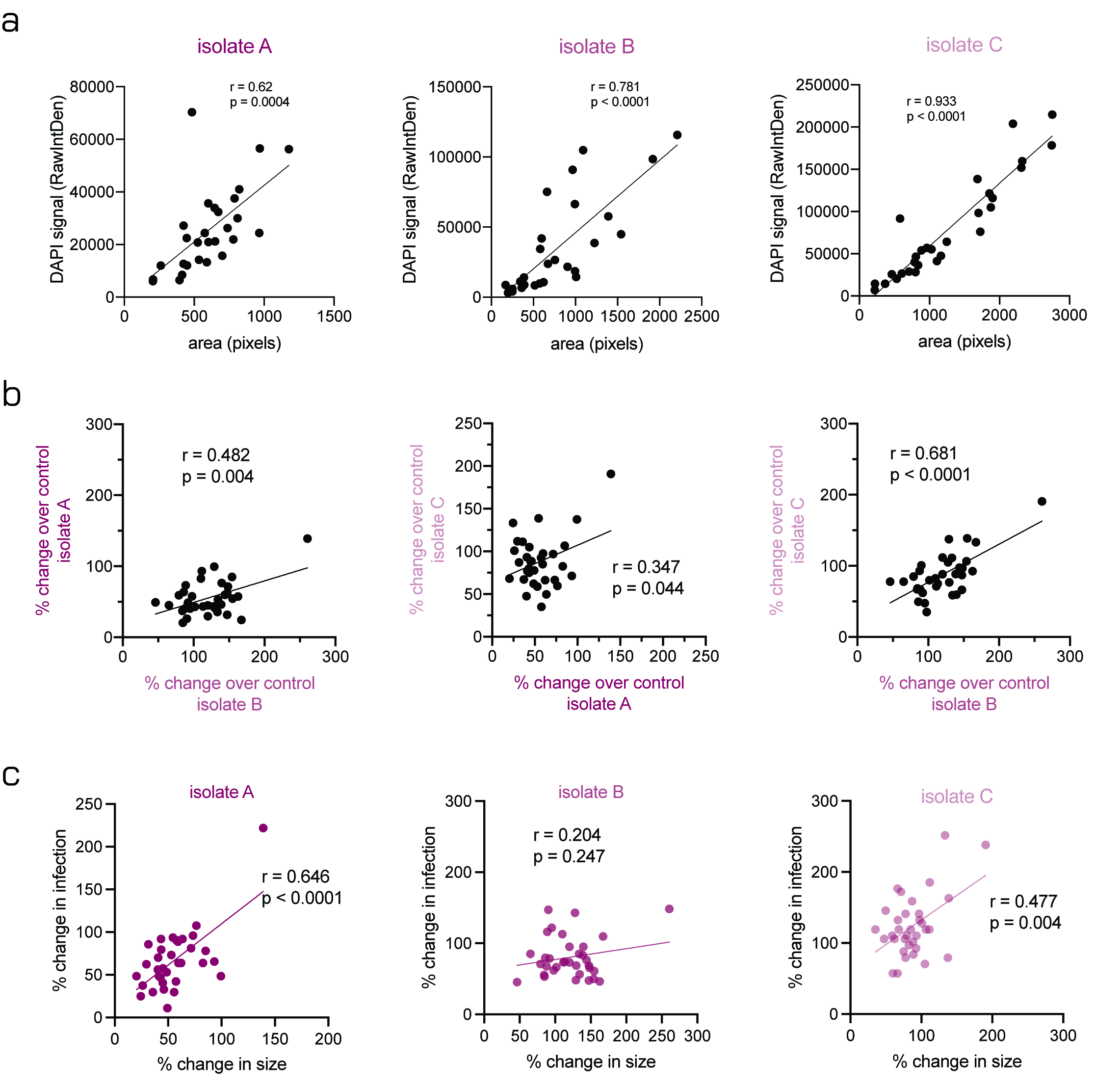

Supplement: S5 Fig — (a) Schizont area, in pixels, plotted against raw internal density (RawIntDen) of DAPI signal within the parasite in control wells. Each dot represents a single parasite from a DMSO-treated well. (b) The mean effect of kinase inhibitors on schizont size plotted for each pair of parasite isolates. Each dot represents a kinase inhibitor. (c) Mean effect of kinase inhibitors on parasite size plotted against mean effect on number of schizonts for each isolate. Each dot represents an inhibitor. Pearson correlation coefficients (r) and p-values (p) were calculated for each comparison. (TIF) [file pntd.0014053.s005.tif]

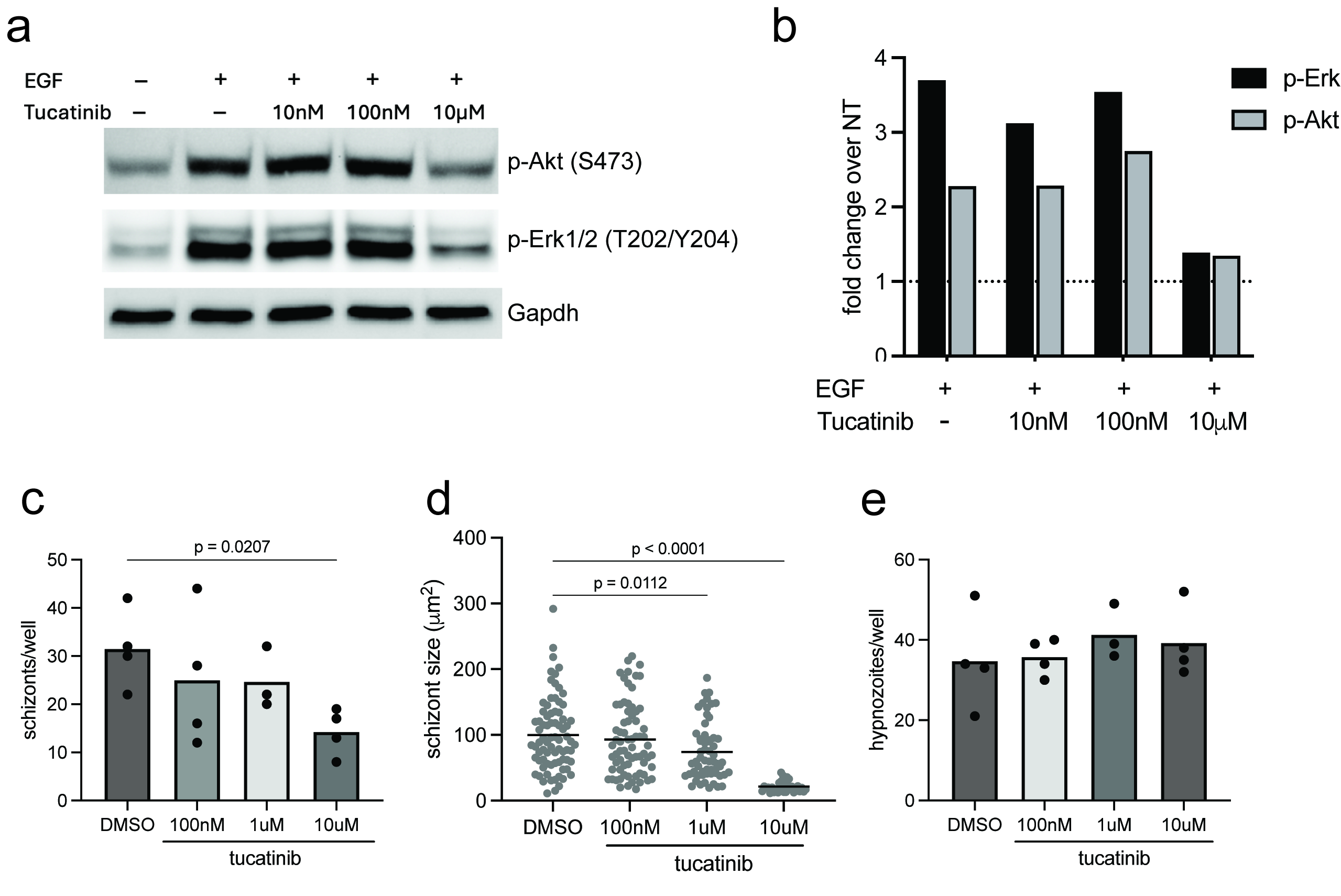

Supplement: S6 Fig — Images (a) and quantification (b) of western blots measuring ERK1/2 and AKT phosphorylation in lysates from uninfected primary human hepatocytes 10 minutes post EGF (10ng/mL) stimulation, with or without a 2 hour tucatinib (10nM-10μM) pre-treatment. Data were normalized to GAPDH loading controls and to the no-treatment (NT) condition. (c) Number of schizonts, (d) schizont size, and (e) number of hypnozoites 5 dpi in parasite isolate E in response to tucatinib treatment (100nM-10μM). Treatment was begun 24 hpi and maintained until fixation. Each dot represents a technical replicate (c, e) or a single parasite (d). Data were analyzed by Kruskal-Wallis test with Dunn’s multiple comparisons test. (TIF) [file pntd.0014053.s006.tif]
