# Supplementary material for: Host kinase regulation of Plasmodium vivax dormant and replicating liver stages
Source: PLoS Negl Trop Dis. 2026 Feb 25;20(2):e0014053. doi: 10.1371/journal.pntd.0014053 (PMC12959719; doi:10.1371/journal.pntd.0014053)
Supplement: S3 Table — Correlation between predicted and tested effects of predicted kinase inhibitors in isolate B. (DOCX) [file pntd.0014053.s009.docx]

|  | | |  | mean % inf | | | | |
| --- | --- | --- | --- | --- | --- | --- | --- | --- |
|  | predicted % inf | | 100nM | 500nM | 1uM | 10uM | max inhibition |  |
| SB203580 | | 70.24 | 84.92 | 69.61 | 65.80 | 35.18 | 35.18 |  |
| JNK inhib V | | 42.99 | 113.79 | 113.79 | 144.83 | 45.97 | 45.97 |  |
| PKR inhib | | 21.93 | 85.61 | 91.53 | 70.35 | 60.18 | 60.18 |  |
| PD169316 | | 47.66 | 119.46 | 85.82 | 68.14 | 75.21 | 68.14 |  |
| p38 Mapk inhib | | 45.22 | 104.57 | 111.39 | 95.47 | 73.88 | 73.88 |  |
| correlation (r) | |  | -0.02641 | -0.4878 | -0.14 | -0.4615 | -0.5185 |  |

**Supplementary Table 3.** Predicted schizont infection rates for five kinase inhibitors based on data from isolate B and mean schizont infection rates in response to treatment with each inhibitor across a range of concentrations. Data are shown as % infection compared to DMSO controls. Pearson correlations (r) were calculated for predicted and actual infection rates at each concentration tested and for the lowest infection rates (maximum inhibition).
