# Supplementary material for: Host kinase regulation of Plasmodium vivax dormant and replicating liver stages
Source: PLoS Negl Trop Dis. 2026 Feb 25;20(2):e0014053. doi: 10.1371/journal.pntd.0014053 (PMC12959719; doi:10.1371/journal.pntd.0014053)
Supplement: S4 Table — Predicted schizont and hypnozoite rates, compared to DMSO controls, in isolates A, B, and C for kinase inhibitors tested in Fig 6a, 6b. (DOCX) [file pntd.0014053.s010.docx]

|  | isolate A | | isolate B | | isolate C |  |
| --- | --- | --- | --- | --- | --- | --- |
|  | | schizont | hypnozoite | schizont | hypnozoite | schizont |
| **PI3-Kg_Inhb** | | **1.73** | **69.93** | **39.19** | **53.6756323** | **84.00039** |
| **CaseinK_II_Inhb_III** | | **17.29** | **53.98** | **35.58** | **34.2204541** | **120.18879** |
| **DNA-PK_Inhb_II** | | **29.99** | 99.62 | **49.34** | **50.5011752** | **88.69064** |
| **PI3-Kg_Inhb_II** | | **33.33** | **79.40** | **42.63** | **32.1415102** | **108.4413** |
| **p38_MAPK_Inhb_III** | | **39.38** | **93.98** | **49.47** | **57.3515645** | **74.55505** |
| LY303511-Neg_control | | 41.92 | 103.33 | 49.77 | 55.6052176 | 85.53282 |
| JNK_Inhb_VIII | | 51.50 | 89.29 | 50.26 | 53.1149832 | 32.50538 |
| Flt-3_Inhb | | 54.84 | 98.52 | 57.91 | 58.1914854 | 82.73329 |

**Supplementary Table 4.** Predicted schizont and hypnozoite infection rates in isolates A, B, and C for kinase inhibitors predicted to reduce infection by >40% for schizonts in two or more isolates and for hypnozoites in at least one isolate. Predictions are shown as percentage infection compared to DMSO controls. Inhibitors in bold were selected for testing in isolate D.
