# Supplementary material for: Host kinase regulation of Plasmodium vivax dormant and replicating liver stages
Source: PLoS Negl Trop Dis. 2026 Feb 25;20(2):e0014053. doi: 10.1371/journal.pntd.0014053 (PMC12959719; doi:10.1371/journal.pntd.0014053)
Supplement: Attachment — Submitted filename: Pv kiR PLoS pathog response to rev.docx [file pntd.0014053.s012.docx]

Reviewer's Responses to Questions

**Part I - Summary**
Please use this section to discuss strengths/weaknesses of study, novelty/significance, general execution and scholarship.

Reviewer #1: The manuscript by Glennon et al. investigates the ability kinase regression to predict host kinases that affect infection rates of the relapsing malaria parasite P. vivax. This builds on previous work where this methodology was used to predict host kinases involved in P. berghei infection. Bringing this methodology to P. vivax requires substantial effort involved with experimental design and access to parasite isolates and the authors are commended for conducting this research. The implications could have tremendous impact in the malaria field by identifying potential pathways that could be perturbed to affect P. vivax infection and would demonstrate kinase regression's utility in another infectious organisms. Yet, the manuscript does not meet the following criteria for publication in PLoS Pathogens: rigorous methodology and substantial evidence for it's conclusions.

Overall the manuscript suffers from rigor and clarity that brings pause to the reader. There are many conclusions drawn and statements of significance, correlation and robustness in the text for which the data is not presented or the data that is presented does not support. The figure legends are incomplete and leave out different parts of the figure (table graphic in figure 3). Additionally, there are presumably typos in the figures as far as kinase inhibitor labels because the figures and the text do not match.

There is no discussion of the main outcome of the manuscript, the inhibitors that were predicted to affect any of the phenotypes, especially those that overlap. There is no discussion of the effect any of these kinases may have on maintaining hypnozoite dormancy or facilitating exponential growth. The hypothesis is presented that these kinases may be involved in growth, but how these kinases could simultaneously stimulate growth and inhibit growth is unclear. Furthermore, validation of a small set of inhibitors needs to be done.

We thank this reviewer for their enthusiasm in applying kinase regression to *P. vivax* liver stage biology. We agree with the reviewer that this effort is extremely challenging. We have, in many places in the revised manuscript, increased the rigor of our reporting, expanding our methods (lines 446-529) and our figure legends (lines 542-654) to provide information about how the experiments were conducted. We have adjusted our language to be more precise about our conclusions, in particular, whether or not correlations are significant (lines 234-246). We have expanded our discussion of the overlap in effect of kinase inhibitors (lines 341-345). We have also provided additional experimental data in the revised manuscript, in which we test additional inhibitors in a fourth parasite isolate (Fig. 6 and Fig. 8, lines 247-259 and lines 301-315).

Reviewer #2: Glennon et al address a significant issue - how host processes might regulate liver infection by P. vivax, the most geographically widespread human-infective Plasmodium species, that also forms dormant liver stages. They focus on the role of host kinase signaling in controlling infection rate and growth of P. vivax liver stages.
Studies of P. vivax are severely limited by lack of experimentally accessible culture systems and the need for fresh patient isolates to initiate mosquito infections. The authors have overcome significant technical hurdles to perform a well-controlled polypharmacology-based kinome screen to identify host kinases that may play a part in regulating P. vivax schizont size, and numbers of schizonts and hypnozoites. The results of the screen are mixed, underscoring the variability between P. vivax patient isolates and the pitfalls of using infections that are not under controlled laboratory conditions.

Kinase Inhibitor Regularization (KiR) analysis has been used previously to identify host kinases involved in regulating Hepa1-6 infection by P. yoelii (same group, Arang et al) and KSHV reactivation (10.1371/journal.ppat.1011169). It can also be used to predict phenotypic effects on untested kinase inhibitors. This study infected the same batch of primary human hepatocytes with 3 different isolates of P. vivax obtained from patients in the Thai-Myanmar border. All 3 isolates expressed the same CS variant but formed schizonts and hypnozoites at different numbers and ratios. All 3 isolates were subjected to a training set of 34 kinase inhibitors to determine effects on schizont size, number and hypnozoite number. Results from the training set were used to predict effects of an additional 178 inhibitors followed by experimental testing of 5 new inhibitors.
Host ERBB2 was predicted to inhibit schizont size, number and hypnozoite number. CSNK2A1 was predicted to regulate schizont and hypnozoite numbers. ACVR1B and OXSR1 were predicted to regulate schizont number and size. CAMK4 and DYRK4 were predicted to regulate schizont size and hypnozoite number. An important caveat is that these predictions apply to specific P. vivax isolates and it is unclear if and to what extent a given kinase’s effect extends to multiple isolates.

Studies were carefully conducted. The authors have done a good job with a technically challenging system. They controlled several variables (same PHH batch, concurrent infections, geographically homogenous isolates, well position). However, the isolates are not clonal or genetically typed. Results of the study offer interesting hypotheses while also, underscoring the highly variable nature of P. vivax liver infection which, in the absence of further experimental validation, makes it difficult to extract clear generalizable conclusions from the current work.

We thank this reviewer for their positive review. Additionally, we are grateful to this reviewer for highlighting the efforts we took to perform a well-controlled screen, despite the difficult experimental system. We have included additional experimental validation for the kinase that is most consistently predicted to have an effect on *P. vivax* liver infection, ErbB2, (Fig. 8) which adds additional evidence for our conclusions.

Reviewer #3: Glennon et al. present an interesting study using kinase regression to uncover critical host kinases for Plasmodium vivax infection. The ability of P. vivax to form dormant hypnozoites is a hurdle to malaria eradication efforts, and this species is a major contributor of disease morbidly. Unfortunately, P. vivax is challenging to study and there is a major gap in knowledge about liver stage schizont and hypnozoite biology which hinders drug development campaigns. The manuscript starts with the demonstration of high variability of 3 parasite isolates in an in vitro model using infection rate, schizont to hypnozoite ratios, and size of the schizonts. Next, a kinase inhibition screen is completed with 28 inhibitors which identified compounds that were consistent across isolates. Of note, it is not possible to know if the observed inhibition is from a host or parasite target. It is shown that none of the kinase inhibitors differentially affected schizonts or hypnozoites, which indicates that the different forms do not significantly utilize different host factors/pathways. Using the power of kinase regression, they analyzed the results from isolate A and predicted the effect of each host kinase on schizonts or hypnozoites. To test some of these predictions, schizont dose response curves during infection were performed with isolate B for 5 kinase inhibitors. Poor correlation of predictions from isolate A are observed on the data gathered from isolate B and C, but there is a higher correlation between the effects on schizonts and hypnozoites from the same isolate. This again highlighted the high variability between different parasite isolates. It is proposed that the high variability in dependency for host kinases could be the origin for heterogeneity observed in P. vivax in the field, but it is likely that many host and/or parasite factors could play a role. Based on these experiments, host kinases that may be important for schizont infection or size, and hypnozoite infection are predicted. This work is a strong starting point for future validation studies.

We thank reviewer 3 for their positive review. We agree with the assessment that “it is likely that many host and/or parasite factors could play a role” in *P. vivax* heterogeneity. We have adjusted our language in the discussion to emphasize that variability in dependency on host kinase signaling may only be a potential contributor to some of this heterogeneity (lines 339-341).

**Part II – Major Issues: Key Experiments Required for Acceptance**
Please use this section to detail the key new experiments or modifications of existing experiments that should be absolutely required to validate study conclusions.

Generally, there should be no more than 3 such required experiments or major modifications for a "Major Revision" recommendation. If more than 3 experiments are necessary to validate the study conclusions, then you are encouraged to recommend "Reject".

Reviewer #1: The manuscript does not meet the following criteria for publication in PLoS Pathogens: rigorous methodology and substantial evidence for it's conclusions. The following modifications and experiments need to be done to meet these criteria:
1. The methods are unclear for a number of analyses and the data are not presented for all of the experiments in which conclusions are drawn. A few examples:
• The rational for the use of a parasite pi4K inhibitor and how it was used is not clear. There are no data presented for this treatment and the concentration of inhibitor used is also not described, yet results are mentioned in the text. Additionally, the inhibitor is known to have off-target host effects and it’s unclear how this many affect any analyses.

We had thought to use the PI4K inhibitor as a positive control for reducing schizont infection but it was not part of the kinase regression or any other analyses in this paper. We have removed it from the paper as it does not add to our study and can lead to confusion.

• No statistics are performed for figure 2. Does the inhibition observed among the inhibitors represent the normal distribution expected under no treatment?

Yes, analysis of variance revealed kinase inhibitor treatment led to a significant difference in mean infection rate than would be expected from random sampling, for all parasite strains and forms. We have added analysis of variance p-values to Figure 2 (figure legend line 560) and updated the text (lines 150-153).

• There is a typo in figure 3 where KI20 is presumably labelled KI27.

We have updated figure 2 where KI27 was mistakenly pattern coded instead of KI20.

• The kinase regression output needs to be represented by a figure in the main text. The table with kinases that are predicted (on what significance level) is not sufficient.

We have created Figure 8 to encompass and expand on the data originally shown in Table 1:

“**Figure 8. Host kinases predicted to regulate *P. vivax* infection.** (a) Overlap in host kinases predicted to regulate schizont numbers, hypnozoite numbers, and schizont size in at least two of three isolates at alpha = 0.8. Kinases predicted to regulate more than one phenotype are indicated bold and color-coded. (b) Number of schizonts, (c) number of hypnozoites, and (d) schizont size 8 dpi in isolate D in response to treatment with tucatinib. (e) Hepatocyte nuclear counts per well normalized to the average of DMSO control wells. Treatment was begun 24 hpi and maintained until fixation. Each dot represents a technical replicate. Data were analyzed by ANOVA with Fisher’s LSD test. * p ≤ 0.05, ** p < 0.01, *** p < 0.0001” (lines 609-617).

• Correlations are presented that are considered significant at some levels yet similar correlations are not considered significant (regardless of p-value) as they are stated in the text.

We apologize for the inconsistency and have adjusted our language to ensure that only correlations with a p-value < 0.05 are referred to as significant. In particular, we adjusted text describing Figure 5 (lines 234-246).

2. Validation with a small set of predicted kinase inhibitors needs to be done.
• The reviewer understands the difficulties of experiments with field isolates, yet treatment with predicted inhibitors and assessing their outcomes are well within the capabilities that the group has already presented. Without validation of a subset of kinases it is difficult to assess the predictive capabilities of the methodology under these variable biological conditions (primary human hepatocytes, two different parasite forms, different parasite isolates).

We have conducted an additional experiment, with a new field isolate independent of the original three isolates used in the initial submission of this manuscript. In this new experiment, we used the chemical inhibitor tucatinib to test the effect of ERBB2 inhibition on schizont numbers, hypnozoite numbers, and schizont size. Importantly, tucatinib is a very specific ERBB2 inhibitor (Kulukian 2020 Mol Cancer Ther, new reference 60) and thus allows us to validate the most consistent prediction from the KiR model with a homologous tool (small molecule compound) as that used to derive the model. We observed an effect on all 3 phenotypes and have integrated these data into the manuscript (Fig 8b-e, Suppl Fig 6, lines 301-315, Suppl File 1). Additionally, we tested the effect of five additional kinase inhibitors which were predicted to reduce schizont and hypnozoite numbers across multiple isolates, (Fig 6a-b, lines 247-259, Suppl Table 4, Suppl File 1), and observed variable results, similar to our original tested inhibitors (Fig. 4c). Taken together, these results are consistent with our original conclusions that while the activity of many inhibitors cannot be accurately predicted due to the variation across field isolates, there are a small subset of kinases (for example, ErbB2) that appear to have more consistent impacts across clinical isolates of *P. vivax*.

Reviewer #2: Results
Main weakness is the lack of experimental validation. Identified host kinases need to be selectively inhibited or knocked down to validate KiR results. Selective inhibitors are available e.g. low dose tucatinib to study ERBB2. ERBB2’s function in KSHV reactivation was recently uncovered using KiR and validated using tucatinib treatment and siRNA knockdown. A dose response curve for tucatinib should be generated to directly test ERBB2’s role in P. vivax LS development.

We have conducted an additional experiment in which we use the chemical inhibitor tucatinib to test the effect of ERBB2 inhibition on schizont infection, hypnozoite infection, and schizont size. We observed an effect on all 3 phenotypes and have integrated these data into the manuscript (Fig 8b-e, Suppl Fig 6, lines 301-315, Suppl File 1). Additionally, we tested the effect of five additional kinase inhibitors which were predicted to reduce schizont and hypnozoite numbers across multiple isolates, (Fig 6a-b, lines 247-259, Suppl Table 4, Suppl File 1), and observed variable results, similar to our original tested inhibitors (Fig. 4c). Taken together, these results are consistent with our original conclusions that while the activity of many inhibitors cannot be accurately predicted due to the variation across field isolates, there are a small subset of kinases (for example, ErbB2) that appear to have more consistent impacts across clinical isolates of *P. vivax*.

In the Discussion section, the authors could consider how often ERBB2 shows up in KiR screens of different phenotypes, as this will indicate if ERBB2 might be a common “false positive” in these kinds of screens.
This is an excellent point. Although ERBB2 does show up as a KiR hit in several published screens it does not seem to be universally predicted. It was predicted to regulate KSHV infection (Olson 2023 PLoS Pathog) and dengue virus infection (Bourgeois 2024 Front Cell Infect Microbiol). However, it is not a predicted kinase for *P. yoelii* LS infection (Arang 2017 Nat Commun) or as a regulator of endothelial barrier integrity (Dankwa 2021 Cell Chem Biol). In the original paper describing KiR, ERBB2 was predicted to regulate cell migration in only 1 of 3 mesenchymal cell line tested (Gujral 2014 PNAS). We have added text to the discussion of the paper (lines 410-412). Additionally, our validation experiment (Fig. 8) using a small molecule inhibitor of ERBB2 that is not included as part of the original screens, lends credibility to the hypothesis that ERBB2 is indeed a regulator of *P. vivax* liver infection and not a false positive.

Fig 4C: The reader has to struggle to understand the experimental setup since the manuscript is fuzzy about important details. What were the selection criteria for the 5 experimentally tested KIs and what are their host kinase target(s)? It is unclear if the 5 KIs were included in the set of 178 KIs whose effects were predicted using the training set data, or were they “new” to the set of 178.

We selected 5 KIs that were predicted to reduce schizont infection by > 80% based on the data from isolate A for further testing. These inhibitors are part of the 178 KIs whose effects were predicted by KiR but were not part of the original screen of 37 inhibitors shown in Fig 2. We have tried to clarify the experimental set-up and selection criteria in the text (lines 206-210).

Given the high level of isolate-to-isolate variation, it is not surprising that KIs predicted using one isolate showed modest activity at best against a second isolate. The more stringent test would be to use the same isolate for prediction and experimental testing. To validate KiR as a useful tool for predicting which KIs might inhibit LS, it is essential to determine correlation between experimental effects of the five new KIs and their predicted results using the same isolate, rather than comparing predictions made using isolate A against experimental results derived from isolate B.

We agree with the reviewer that comparing predictions and experimental results within the same isolate is a good validation for KiR. We have added a supplementary table (Supplementary Table 3, lines 215-223) comparing predicted and measured inhibition of schizont infection for the five inhibitors for which we have data. We have not found that KiR drug predictions are particularly accurate as to the exact magnitude of inhibition of a phenotype and do not see a strong correlation between predicted and measured values. This could be influenced by the low sample size and the compressed range of values. It is also very possible that KiR drug predictions are not very robust in this system, possibly due to factors such as bioavailability. This possibility is supported by the shift of IC50s to the right seen for most inhibitors. There is also the potential for compounded error as the algorithm bases its drug predictions on the kinase predictions.

Table 1 Legend. What is meant by ‘Overlapping kinases’?

We have edited the text in the legend, replacing ‘overlapping kinases’ with ‘kinases predicted to regulate more than one phenotype’ (lines 611-612).

Were the 3 isolates genotyped for MSP-1? Previous results suggested that isolates expressing the same CS variant have similar hypnozoite:schizont ratio but authors did not observe this.

Unfortunately, the isolates were not genotyped for MSP-1. We have added a line discussing this important point on lines 430-431.

Discussion:
A few assertions lack references, for eg.
Even within sporozoites isolated from a single mosquito infection…
Machinery that underlies hypnozoite formation also underlies schizont growth
We have gone through the text to clarify and provided references for assertions (lines 34, 317-321) or reworded them to make it more clear what is speculation (lines 376-378).

Discussion lacks comparison of results obtained by the same ground using KiR in P. yoelii. Presumably host kinases that regulate LS infection/growth in both species may be the more robust targets. In addition, their role can be rigorously evaluated using siRNA, genetic knockdowns etc in the rodent system. The top candidate in this screen, ERBB2 did not appear in the P yoelii KiR screen. But as reported above, it is found to play an important role in KHSV reactivation.
We apologize for this oversight. We do see some overlap in kinases predicted to regulate schizont numbers and size with those predicted to regulate *P. yoelii* infection. However, our concern in relying too heavily on this overlap for follow-up is the potential confounding effect of host cell type. Our vivax infections are conducted in primary human hepatocytes while the *P. yoelii* infections were conducted in mouse hepatoma cells (hepa1-6). We hypothesize that the difference in host environment between primary hepatocytes and hepatoma cells may heavily influence host kinase dependencies and the effects of kinase inhibitors. Though beyond the scope of this study, a comparison between the two parasites in the same host cell background could be useful for future validation. We have added text to the discussion to address this topic (lines 404-412).

Materials & Methods
Description of treatments and IFA conditions lack important detail. For eg, concentrations of 178 inhibitors and antibody source and concentrations used are not mentioned.

We have added additional details to our methods, including the concentrations of inhibitors and the source and concentration of antibodies used in IFAs (lines 464-472).

Reviewer #3: The work would be strengthened with studies to validate the importance of a predicted host kinase in schizont infection or size

We have conducted an additional experiment in which we use the chemical inhibitor tucatinib to test the effect of ERBB2 inhibition on schizont infection, hypnozoite infection, and schizont size. We observed an effect on all 3 phenotypes and have integrated these data into the manuscript (Fig 8b-e, Suppl Fig 6, lines 301-315, Suppl File 1). Additionally, we tested the effect of five additional kinase inhibitors, which were predicted to reduce schizont and hypnozoite numbers across multiple isolates, (Fig 6a-b, lines 247-259, Suppl Table 4, Suppl File 1).

**Part III – Minor Issues: Editorial and Data Presentation Modifications**
Please use this section for editorial suggestions as well as relatively minor modifications of existing data that would enhance clarity.

Reviewer #1: There are a fair amount of minor issues with this manuscript, for brevity, some are summarized here:
1. Please add line numbers.

We have added line numbers.

2. The term phenotype is used to interchangeably refer to phenotypes observed in the field and phenotypes of field isolates observed in the lab. Hypnozoite ratios cannot be observed in the field, they can only be observed using field isolates in laboratory experiments. Phenotypes of parasites in the field include relapse frequency and time to recurrence.

We apologize for the sloppy language and have edited the text to accurately describe observable field vs lab phenotypes (lines 14-15 and 612-616).

3. The word infection is used in many circumstances to describe the infection rate. The phenotype that is quantifiable and that is being observed is the infection rate. The process of establishing infection is not being investigated.

We agree with the reviewer and have updated our language throughout the text to use “number of schizonts/hypnozoites” in place of “infection.”

4. There are no figure callouts to give figure 2 context. There is only one callout at the end of three paragraphs discussing the figure.

There are references to figure 2 on lines 146 and 183 of the results section. We have added an additional callout on lines 150 and 152-153.

5. The manuscript does not discuss that the timepoint of drug treatment is 24 hours post-infection, which is when the parasites cannot be distinguished between schizonts and trophozoites. The abstract also alludes that the parasite enters the cell as a committed schizont or hypnozoite which is not the case. The timing of drug treatment and the effects it could have on conclusions also be explored in the discussion.

We have added discussion on the timing of drug treatment and what questions remain regarding how the stage of parasite development may lead to differences in effects of treatment to the discussion (lines 396-403).

6. The figure legends lack details and explanation of all content of the figure. Abbreviations are not defined and colors used in figures are not defined.

We have added additional details to figure legends including defining all abbreviations and color coding.

7. There are no methods for dose response experiments (compound treatment etc.) and no curves fit to the data.

We have added details on dose response experiments to the methods section (lines 465-468). We do not show curves calculated to fit the data as the large majority of compounds tested do not show a gradual dose response and did not display a standard slope. We have reviewed the text to ensure we do not make any quantitative claims about precise levels of predicted inhibition or EC50s.

8. Supplementary figure legend 3 states KI41 in the legend, but the graph represents each inhibitor?

We apologize for the typo. Each dot represents a single inhibitor from the screen. The legend has been corrected (lines 629-633).

9. There is no mention in the discussion of what impact this study could have on malaria.

We apologize for this oversight and have added the following text to the discussion: (lines 413-420).

Reviewer #2: P3: Typo ‘Mayanmar’ border
Thank you for catching this typo. The text has been corrected to spell Myanmar correctly (line 62).

Clearly state whether or not the same batch of PHHs was used throughout the study or only for experiments performed in Fig 1? If different PHH batches were used in the study, then it should be presented as a confounding variable.

The same lot of primary hepatocytes was used for all experiments conducted in this paper. We have tried to clarify this in the results and methods sections (lines 111-112, 459-460).

P6: ambiguous sentence: unclear what is meant by if schizonts were “more susceptible”. Do the authors mean if schizont number is more malleable and/or schizont size? Reword sentence for greater clarity.

We have edited this sentence to indicate we are talking about the number of schizonts (lines 161-162).

P9: ambiguous sentence: “…distinct growth factors influence infection maintenance and parasite growth”.
How do the authors define infection maintenance – is it the number of LS over time, in a given culture? If so, then how can infection maintenance be assayed when LS were quantified only on d8? Similarly, what is the measure of “parasite growth” – schizont size or # of parasite nuclei per schizont?
We have edited the text to clarify (lines 281-282): we are hypothesizing that different host factors may regulate the presence/absence of parasites vs the size of parasites. We do have evidence showing schizont size correlated with parasite nuclear stain signal, suggesting size may be indicative of parasite biomass (lines 267-269, Suppl Fig 5a).

Manuscript refers repeatedly to schizont infection and hypnozoite infection giving the impression that schizonts and hypnozoites are the infectious agents rather than the consequence of infection by sporozoite. I suspect that what the authors really mean is number of schizonts and number of hypnozoites. If so, it would be best to use these terms.
We agree with the reviewer that “number of schizonts/hypnozoites” is more accurate and have updated our language throughout the text.

Results should state KIs were added 24h pi, to ease the burden on the reader for finding this information.

We have added this information to the results section (lines 137-138, 211, 254).

Reviewer #3: Figure 5b – why where hypnozoite predictions from isolate A compared and plotted against the drug screen in isolate C? Based on figure 1, it appears that isolate C was deficient in hypnozoites. If this is true, would it be better to compare the predictions from isolate A and the effect seen in isolate B?

Thank you for catching this typo. The comparison was between the predictions from isolate A and the screen in isolate B and we have updated the axis in Figure 5b to reflect this.
