# Supplementary material for: Host kinase regulation of Plasmodium vivax dormant and replicating liver stages
Source: PLoS Negl Trop Dis. 2026 Feb 25;20(2):e0014053. doi: 10.1371/journal.pntd.0014053 (PMC12959719; doi:10.1371/journal.pntd.0014053)
Supplement: Attachment — Submitted filename: PLoS NTD reviewer response_updated.docx [file pntd.0014053.s013.docx]

Reviewers' comments:

All line references are for the marked version of the manuscript.

Reviewer's Responses to Questions

Reviewer #1: -Are the objectives of the study clearly articulated with a clear testable hypothesis stated?

yes

-Is the study design appropriate to address the stated objectives?

Positive control are missing when addressing KI effect on the schizonts. I think it would be important to explain why the number of repetition (wells) isn’t the same for each conditions of figure 1. Authors should indicate if sporozoite viability have been tested or not

We agree with the reviewer that a schizonticidal positive control is important to this study. The phosphatidylinositol 4-kinase inhibitor, MMV390048, was used as a positive control for schizont inhibition in parallel studies conducted with these parasite isolates (same time, same vial of primary human hepatocytes) that have already been published (Rodriguez-Hernandez et al. 2023 Nat Commun). These data were included in an earlier version of the paper, but we did not want to duplicate already published data and removed them during the review process. Instead, we have now added a reference to them in the results section (lines 161-164).

For each experiment we used multiple control wells spread throughout the 384-well plate so that we would be able to have sufficient controls for comparison in the case of technical error or contamination. This number varied across experiments based on cell and sporozoite numbers. We chose to comprehensively plot all the data we had, even if it meant uneven numbers across isolates. We have added this information to the methods section (lines 490-491, 505-506).

All infections were conducted immediately after dissection of salivary glands and isolation of sporozoites from live mosquitoes within a similar time frame post-feeding, therefore we do not anticipate major differences in parasite viability between isolates due to handling. Infectivity of primary hepatocytes was measured for all strains 8 dpi in control wells (Figure 1). An option for future studies would be to measure hepatocyte invasion rate which could have varied among isolates. We would anticipate that differences in invasion rate would be captured in infection data 8 dpi, however we cannot deconvolve differences in invasion vs infection maintenance in this study. We have added text stating this to the study limitations section (lines 454-457).

-Is the population clearly described and appropriate for the hypothesis being tested?

Yes

-Is the sample size sufficient to ensure adequate power to address the hypothesis being tested

yes

-Were correct statistical analysis used to support conclusions?

yes

-Are there concerns about ethical or regulatory requirements being met?

I haven’t noticed ethical concerns

Reviewer #2: Yes

Results

Reviewer #1: -Does the analysis presented match the analysis plan?

yes

-Are the results clearly and completely presented?

yes

-Are the figures (Tables, Images) of sufficient quality for clarity?

yes

Reviewer #2: Yes

Conclusions

Reviewer #1: -Are the conclusions supported by the data presented?

yes

-Are the limitations of analysis clearly described?

Yes

-Do the authors discuss how these data can be helpful to advance our understanding of the topic under study?

yes

-Is public health relevance addressed?

yes

Reviewer #2: Yes

Editorial and Data Presentation Modifications?

Use this section for editorial suggestions as well as relatively minor modifications of existing data that would enhance clarity. If the only modifications needed are minor and/or editorial, you may wish to recommend “Minor Revision” or “Accept”.

Reviewer #1: L183 perhaps recalcitrant isn’t the most accurate term, I would keep it simple an say “not susceptible” or similar.

We have updated the text to replace “recalcitrant to” with “not responding to” (line 197).

Reviewer #2: (No Response)

Summary and General Comments

Use this section to provide overall comments, discuss strengths/weaknesses of the study, novelty, significance, general execution and scholarship. You may also include additional comments for the author, including concerns about dual publication, research ethics, or publication ethics. If requesting major revision, please articulate the new experiments that are needed.

Reviewer #1: Glennon et al. investigated the dependencies of P. vivax liver stages (schizont, hypnozoite) to host phosphosignaling pathways through evaluation of kinase inhibitors. To carry out their investigations the authors used a LS culture system based on human primary hepatocytes and imaging post UIS4 marking (fluorophore coupled) as readout. This methodology was proved to be adequate in multiple studies. Unfortunately, these results are based on a limited number of isolates but this has to be put in perspective with the low P. vivax incidence today in south east Asia and the demanding aspect of Pv LS culture. The authors also present limitations, I essentially agree with, of their study.

Results

Authors presented first results of hypnozoite production rate and number of parasites. These variables vary in a certain extent according to the isolate of origin. This finding is not particularly innovative. In addition, the variable number of well analyzed per isolates, the large range of value obtained is, to me, making conclusions out these experiments a bit uncertain (see results comments) even if those are to me consistent with the literature. There is from my perspective a limited added value to have these results presented here, perhaps SI section would be more suitable. Then the authors present results based on host kinase inhibition vs. hypnozoite and schizont numeration. ¾ isolates collected were used to do so. The results about how some specific host KI might influence the development of the parasite is promising although deeper investigation appear necessary. The conclusion is based on an association and extensive further biochemical measurement would be necessary. Using these results, the authors modelized the activity of a larger pool of KI reaching 178 potential drugs presenting an effect. Out of these 178 drugs, 5 were to my understating actually verified in the LS model. The conclusion is a certain weakness of KIR model to predict the activity with an attributed major impact of strain variability unlike data get pooled. Despite the interest of this part, I’ve found the absence of specific schizont inhibitor control very detrimental. Finally, the authors concertized their investigation on host kinase through evaluating the impact of ERBB2 inhibition, previously identified as potentially favoring parasites development. Impact on schizont looks clear and dose dependent unlike what has been observed with hypnozoite. Here again a positive control would have been welcome.

We agree with the reviewer that a schizonticidal positive control is important to this study. The phosphatidylinositol 4-kinase inhibitor, MMV390048, was used as a positive control for schizont inhibition in parallel studies conducted with these parasite isolates (same time, same vial of primary human hepatocytes) that have already been published (Rodriguez-Hernandez et al. 2023 Nat Commun). These data were included in an earlier version of the paper, but we did not want to duplicate already published data and removed them during the review process. Instead, we have now added a reference to them in the results section (lines 161-164).

L117 Why the number of sporozoites isolated out of isolate A isn’t mentioned?

Unfortunately, the parasite count per mosquito for isolate A was not recorded. We have added a line to the text stating this (line 130).

Fig1b/c, it is not clear to me why isolates data appeared to be based on a different number of wells? Higher wells numbers ie in A and D gave higher value spread (which is normal) then it don’t really sounds to me fully comparable with B and C as higher wells number might change the conclusion.

For each experiment we used multiple control wells spread throughout the 384-well plate so that we would be able to have sufficient controls for comparison in the case of technical error or contamination. This number varied across experiments based on cell and sporozoite numbers. We chose to plot all the data we had, even if it meant uneven numbers across isolates. We agree with the reviewer that this could bias any comparisons of the range of parasite numbers/well across isolates and have reviewed the text to be sure we do not make any claims about the spread of parasite numbers per well (lines 137-144).

L182 the observation of concomitant reduction of schizont and hypnozoite numbers unlike data generated by other groups with specific schizonticides can also be indeed a matter of specificity and might also be the reflect of the inherent toxicity/metabolism alteration of the KI to the host cells.

We agree with the reviewer that the increased sensitivity of hypnozoites to some kinase inhibitors compared to schizonts could be due to either the effects of the inhibitor on a particular protein/signaling pathway or due to larger scale alterations made to the host hepatocyte. We removed inhibitors from our screen that significantly reduced primary hepatocyte numbers (Suppl Fig 1, lines 153-155) in an attempt to minimize the impact of more global toxicity on our results. An interesting hypothesis for future consideration would be that replicating schizonts are more actively altering the host hepatocyte, making it more resistant to metabolic changes or toxicity upon kinase inhibitor treatment.

Discussion

L325: the data presented are not consistent with the variable reactivation temporality observed in the different geographic settings. Authors should rephrase by mentioning only the infection rate aspect.

We have edited the text to avoid the implication that our data are consistent with the geographical variability of reactivation (lines 343-344).

L434 The “origin” of a given P. vivax isolate ie relapse or de novo infection is indeed particularly complex to determine but does this represent a variation factor more than parasites population heterogenicity? To reinforce this statement a citation would be welcome.

We agree with the reviewer that population heterogenicity is a much more likely source of variation and we have not found conclusive evidence in the literature that heterogeneity is significantly different between de novo and relapsing infections. We have updated the text to reflect this (lines 450-452).

The authors should also discuss the drugging timing, in the present design they saw variable activity by adding drug at 24h PI. Effect or not on later parasites should be discussed.

Text discussing the timing of drug treatment and the effect on parasites is in the discussion (lines 418-425).

Methodology

CSP typing is presented in the manuscript as limited (I agree with this statement) so finally why using such approach especially when non-confidential approaches such as whole genome sequencing or targeted amplicon sequencing exist and would be more informative.

We agree with the reviewer and plan to more fully characterize parasite isolates used in the future. Unfortunately, we were unable to sequence the parasites that were available for this study.

For the infection the authors standardize their inoculum to 14K sporozoites/well but does sporozoites viability for each batch has been verified?

All infections were conducted immediately after dissection of salivary glands and isolation of sporozoites from live mosquitoes within a similar time frame post-feeding, therefore we do not anticipate major differences in parasite viability between isolates due to handling. Infectivity of primary hepatocytes was measured for all strains 8 dpi in control wells (Figure 1). An option for future studies would be to measure hepatocyte invasion rate which could have varied among isolates. We would anticipate that differences in invasion rate would be captured in infection data 8 dpi, however we cannot deconvolve differences in invasion vs infection maintenance in this study. We have added text stating this to the study limitations section (lines 454-457).

Reviewer #2: To learn whether responses to host (human) kinases govern schizont/hypnozoite growth (and therefore relapse patterns) in vivax parasites, Glennon and colleagues use 4 P. vivax isolates from NW Thailand of the same CSP variant to infect liver cells from the same primary donor and attempt to discover kinases that alter parasite schizont and hypnozoite numbers and sizes in the same way across isolates. Unfortunately, variation in infection phenotype and responses to kinases between isolates makes it hard to pinpoint any common host kinase pathways or kinase inhibitors that consistently/predictably affect the parasite phenotypes investigated. In particular, the authors were limited to two robust isolates (A&B), with C infecting low numbers of cells, and isolate D saved for replication experiments, which as a whole, did not yield robust conclusions or targets. The authors were forced to test inhibitors that acted similarly in 2 of 3 isolates. Since the six kinase inhibitors tested for validation all reduced schizont size at the highest concentration, I am not sure if the prior work was indeed predictive, or if many inhibitors are just toxic at high concentration.

The authors conclude that better characterization of the genetic diversity of isolates used (and therefore ability to select genetically similar isolates to probe) may help reduce the variability in their outcomes. It is true that even a single “isolate” with the same broad CSP variant identified by PCR (RFLP) might actually contain different parasite variants (“strains”) arising from separate hypnozoites and this within-host genetic diversity could lead to a spectrum of phenotypic outcomes even within the same isolate. Also, it is hard to ignore the extent of global genetic diversity of P vivax (S American vs South Asian vs SE Asian isolates), making even robust outcomes in a genetically homogenous groups of isolates hard to translate more widely. I agree with a prior reviewer that the work is a good starting point, and hopefully the authors can further investigate their subset of kinases in a (much) larger, more diverse set of clinical isolates (that includes subsets of genetically similar isolates), while also thinking about the effects of kinase inhibitor bioavailability and epigenetic variation on their experiments.

Though I have no firsthand experience with this type of experimental work, I agree with the prior reviewers that the experiments involve high technical difficulty, and at least in the revised version, the authors go through pains to clearly explain their results and next steps through multiple figures and tables. In response to previous reviewers, the authors have provided further validation of their conclusions by using tucatinib to test the effect of ERBB2 inhibition (as well as 5 other kinase inhibitors) on schizont/hypnozoite numbers and size, with some predictable effect on these phenotypes - mostly reducing schizont numbers in a dose response patterns, in two different isolates.

The paper underwent a thorough review at PLoS Pathogens, and the authors appear to have taken a rigorous approach to addressing those previous comments and feedback.

Minor comments:

Line 72 – “blocking apoptosis”  blocking of apoptosis

This correction has been made.

Line 213 – “only two of the five inhibitors, SB203580 and JNK inhibitor V, significantly reduced schizont numbers in isolate B at any of the concentrations tested (Fig. 4c)” In Fig 4C, JNK inhibitor V reduced schizont numbers at 10uM, but increased schizont numbers at 1uM. It is odd to single this out given the dose response design. I might only highlight the first inhibitor which achieves a clear dose reponse effect. PKR inhib looks look it might show a dose response effect if more replicates were done.

We agree with the reviewer that focusing on SB203580 and its dose response is a better representation of the data in figure 4c and have adjusted the text (lines 228-231).

On a related note, the effects noted in Fig 6 all occur only at 10uM, which the authors might comment on in their discussion.

We have adjusted the text to make this point clear (lines 270-272).
