# Supplementary material for: Host kinase regulation of Plasmodium vivax dormant and replicating liver stages
Source: PLoS Negl Trop Dis. 2026 Feb 25;20(2):e0014053. doi: 10.1371/journal.pntd.0014053 (PMC12959719; doi:10.1371/journal.pntd.0014053)
Supplement: Attachment — Submitted filename: PLOS ntd resp to reviewers 12-11-25.docx [file pntd.0014053.s014.docx]

**Journal Requirements:**

1) We have noticed that you have a list of Supporting Information legends in your manuscript for S1 Figures to S5 Figures. However, there are no corresponding files uploaded to the submission. Please upload them as separate files with the item type 'Supporting Information'.

We apologize for the error, Figures S1-S5 have been uploaded in the resubmission.
 
Note: If the reviewer comments include a recommendation to cite specific previously published works, please review and evaluate these publications to determine whether they are relevant and should be cited. There is no requirement to cite these works unless the editor has indicated otherwise.

**Reviewers' comments:**

Reviewer #3: Glennon et al. present an investigation into the role of host phosphosignaling during Plasmodium vivax liver stage infect, aiming to elucidate how these pathways influence parasite development, including hypnozoite formation and schizont development. The manuscript provides evidence that host hepatocyte kinase signaling contributes to liver stage biology, while acknowledging several limitations that are appropriately discussed. The authors have addressed prior reviewer comments effectively; however, the following points require clarification before acceptance.

1. Inclusion of MMV390048 Data- Incorporating MMV390048 data alongside the current dataset would enhance interpretability. While concerns about duplicating data are valid, proper citation and clear indication in the results should mitigate this issue. Including these data would allow readers to more easily assess hypnozoite and schizont observations and help with navigating the interpretations being drawn.

We have added the level of inhibition with MMV390048 treatment to Figure 2 as a red dashed line (Fig 2, Figure legend lines 600-601)

2. Measurement Approach - Please clarify the rationale for using parasite diameter rather than area in the analysis of P. vivax liver stages to classify hypnozoites versus schizonts. Generally, area is used.

When screening parasites for categorization as schizont vs hypnozoite we used diameter as an initial factor, due to it being faster to determine and then, more critically, we used DAPI staining to distinguish parasites with single vs multiple nuclear foci. All measurements of size in figures are of parasite area (Fig 1d, Fig 7, Fig 8d).

3. VK210 Variant - The manuscript notes parasite-intrinsic factors; however, it would be valuable to discuss how these findings confirm or challenge previous data on VK210, given that all parasites studied are VK210 variants.

In the discussion we state that our results are consistent with the work of Vantaux et al. 2022 who also saw significant variation in infection phenotypes across parasite isolates. This paper does not state which variant it used, however we assume it is likely VK210 as well. Mikolajczak et al. 2015 compared HuHep FRG mouse infection rates among 3 isolates each of VK247 and VK210 and between two hepatocyte donor lots infected with the same isolate. The heterogeneity in VK210 isolate infection rate (3-4x difference) is consistent with our findings. They did not observe differences between the two hepatocyte donor lots (Figure S5). We have added a sentence describing this to our discussion (lines 357-361). To the best of our knowledge these are the only published studies that compare P. vivax isolates in the context of liver-stage infection with a sample size conductive to drawing any conclusions. Two single cell transcriptomic studies of *P. vivax* LS infection each used 2 patient isolates which either were pooled (Mancio-Silva 2022) or not compared statistically (Ruberto 2022).
4.Assessment of Hepatocyte Viability - The authors state that toxic compounds were removed, but was hepatocyte viability assessed (e.g., via hepatocyte nuclei counts)? If performed, please indicate where this is reported; if not, consider adding this analysis to confirm that toxicity does not confound the results.

Toxicity was evaluated by hepatocyte nuclei counts and shown in Suppl Fig 1 and in Fig 8e. Please see lines 156-159 in results section, lines 516-520 in the methods, lines 657-659 in the figure legends, and lines 1026-1030 in the supplemental figure legends.

5. Interpretation of Figure 2- How do the authors reconcile slowed schizont growth with changes in hypnozoite numbers? Could some parasites classified as hypnozoites represent slowed schizonts? Addressing this possibility earlier would help bridge gaps left by missing controls noted by other reviewers.

There is currently no distinction between a hypnozoite and a schizont that has stalled before its first DNA replication. Because we only classified parasites with a single nuclear foci as hypnozoites, we do not think these are schizonts that began development and then stalled. However, we do not know if smaller schizonts are parasites that began replicating and then slowed, began replicating later, or maintained a steady but slower replication rate. This would require a time course or live-imaging readout that is beyond the scope of this study. We have added a line discussing this to our study limitations section (lines 465-467).

6. The statement “we observe a strong positive correlation between the impact of a given inhibitor on schizonts and hypnozoites…” appears to suggest hypnozoites are unaffected by kinase manipulation, yet other sections imply otherwise. Please clarify this point. Additionally, since hypnozoites increase in size between days 3 and 8, was size shifting analyzed within this population?
A correlation between the effect of an inhibitor on schizonts and hypnozoites does not imply that hypnozoites are not affected by kinase manipulation. We have modified the text to try to clarify our statement (lines 207-211): “we observe a strong positive correlation between the impact of a given inhibitor on schizonts and hypnozoites. This suggests that drugs that inhibit schizonts are also likely to inhibit hypnozoites. The underlying biological origins of this phenomenon remain unknown, but it does suggest that the targets of the most effective inhibitors are relevant for both schizont growth and hypnozoite survival.”

We did not measure hypnozoite size in our experiments. We were concerned that the level of resolution at which we imaged infected plates did not allow for accurate measurement at this scale.

7. Normalization in Figure 4 -- Why were parasite counts presented per well rather than normalized to controls with known outcomes on Pv liver stages? For example, normalization to monensin or nigericin (which eliminate all parasite forms) would add rigor compared to raw counts.

We used a diluent control (DMSO) for all statistical analysis of drug treatments. We did not use any other control drug treatments as any potential variation in response across replicates could have added an additional confounding factor to data normalization. In figure 2 we normalized parasite counts to the average of the DMSO control to facilitate comparisons across replicates with varying infection rates (Raw counts are available in supplementary files). In figures that only utilize a single isolate (including figure 4) we show raw parasite counts as we believe this provides more information/transparency for the reader, particularly the intensity of infection in the absence of treatment for the particular isolate being used for each experiment.

8.The manuscript states that the data explain 'heterogeneous outcomes observed with P. vivax isolates', but how the data actually contribute to this is unclear. The results primarily indicate that in vitro perturbations occur, likely influenced by cell type choices, as noted by the authors. Please clarify how the presented data support this conclusion.
We are not entirely clear what the reviewer means by “in vitro perturbations” occurring “influenced by cell type choices”. What we suggest is that the difference we observe in how parasite isolates, within a single variant, respond to manipulation of host phosphosignaling could contribute to the heterogeneity of *P. vivax* phenotypes in the field, such as the rate and timing of relapse (within the larger patterns already described between variants and geographical regions). We have attempted to clarify this point in the discussion (line 371-372).

9. When kinase treatment strongly correlated with schizont inhibition, did it also affect hypnozoite size, or was growth slowing exclusive to schizonts?

We did not measure hypnozoite size in our experiments. For future work, we would be very interested in measuring the effects of kinase inhibition on hypnozoite phenotypes that are relevant to infection dynamics in the field including longer term maintenance and re-activation.

**Figure resubmission:**

While revising your submission, we strongly recommend that you use PLOS’s NAAS tool (https://ngplosjournals.pagemajik.ai/artanalysis) to test your figure files. NAAS can convert your figure files to the TIFF file type and meet basic requirements (such as print size, resolution), or provide you with a report on issues that do not meet our requirements and that NAAS cannot fix.

After uploading your figures to PLOS’s NAAS tool - https://ngplosjournals.pagemajik.ai/artanalysis, NAAS will process the files provided and display the results in the "Uploaded Files" section of the page as the processing is complete. If the uploaded figures meet our requirements (or NAAS is able to fix the files to meet our requirements), the figure will be marked as "fixed" above. If NAAS is unable to fix the files, a red "failed" label will appear above. When NAAS has confirmed that the figure files meet our requirements, please download the file via the download option, and include these NAAS processed figure files when submitting your revised manuscript
